# Negative impact of the COVID-19 pandemic on the management of cervical cancer patients in Zambia

**DOI:** 10.3332/ecancer.2020.ed103

**Published:** 2020-07-07

**Authors:** Dorothy Lombe, Misheck Phiri, Susan Msadabwe

**Affiliations:** 1 Cancer Diseases Hospital, Department of Oncology, Lusaka, 10101, Zambia; 2 Cancer Diseases Hospital, Department of Radiation Therapy, Lusaka, 10101, Zambia; a http://orcid.org/0000-0002-5083-1801

**Keywords:** brachytherapy, COVID-19, cervix, cancer

## Abstract

International travel has largely been suspended due to the COVID-19 pandemic. Due to this situation, Zambia has not been able to import radioactive isotopes for High Dose Rate (HDR) brachytherapy, Iridium 192 (I192) and this has led to suspension of treatment for patients. Cancer of the cervix is the most common cancer in Zambia and brachytherapy is a core component of the treatment armamentarium. Mitigation strategies may include external beam radiotherapy boost or hysterectomy but both systems are overburdened and fragile.

The COVID-19 pandemic has demanded a shift in practice and social norms throughout the world and across disciplines. Zambia, a country in the south-central part of Sub-Saharan Africa with a population of approximately 17 million, took pragmatic steps to continue providing cancer care and utilised the positive changes to enhance service delivery to oncology patients as outlined in our previous publication [1].

The burden of SARS-CoV-2 infection in Zambia has not been as heavy as that seen in some countries in the northern hemisphere. Currently the total number of cases confirmed is 1477 with only 246 active [2]. The total number of deaths is 18, all of whom had severe underlying co-morbidities. One thousand two hundred and thirteen people have recovered and discharged from quarantine centres. Despite the positive shifts in practice experienced broadly during this pandemic across the sole national cancer centre, the brachytherapy service in Zambia has been negatively impacted due the inability to import radioactive isotope sources as a secondary effect of suspended international travel.

Zambia utilises high dose rate (HDR) brachytherapy with an Iridium 192 (I^192^) isotope (half-life of 90 days). This warrants a source change, which is imported from abroad, every 3 months. When the dose rate is too low due to delayed source change, an interlock is activated leading to failure to treat any patients. This interlock for the system in Zambia occurred on 2 June 2020. For the month of June, 28 cervix cancer patients are not going to complete their course of radiotherapy (external beam and brachytherapy) within the recommended 8-week treatment period. If this persists into the following month approximately a further 45 women will have a high likelihood of uncontrolled cervical cancer and poor outcomes for a highly curable disease in the right conditions.

Suspension of brachytherapy services is catastrophic on the overall cancer outcomes as Zambia has one of the highest global incident rates of cervical cancer at 66.4 per 100 000 and death rate of 1 839 per year [3]. Cervical cancer constitutes approximately 25% of all new cancers incident per year with 90% of the cases being locally advanced [4]. Brachytherapy is a key component of the treatment armamentarium against locally advanced cervical cancer as it increases the ability to deliver the high doses of 85-90 Gy in total needed for cure whilst minimising organ at risk (OAR) dose [5–7]. Although it has been proposed as an alternative in the absence of brachytherapy, external beam boosts, especially with the quality of photon radiotherapy available in Zambia, is likely to significantly reduce the chances of good outcomes for this disease [8].

Additionally, brachytherapy is part of the standard of care as per indication for adjuvant treatment of endometrial cancer either as a sole modality or as a boost. In recent times, the brachytherapy service at the Cancer Diseases Hospital (CDH) has expanded to include organ-preservation treatment of penile cancer that is endemic in this region [9].

At the beginning of the pandemic the staff in the brachytherapy section took bold steps to maintain the pace of treatment of 10 patients per day by adjusting the work schedules and shifts. The longer treatment times (1 hour 14 minutes for a 7 Gy plan) per patient were taken in their stride and seen as an opportunity to ensure increased uptake of 3 dimensional planning as time in between patients allowed for the steps of CT simulation, contouring and planning. The interlock of the system however, has put an indefinite halt to the hope of curative treatment for a significant number of Zambian women, as the build up of a backlog will be rapid and large.

The alternative options of external boost or hysterectomy have been proposed but both the external beam and surgical systems are over burdened already [8, 10]. Currently the waiting time for both external beam radiotherapy and surgery is 4 months. Careful planning will need to be done to avoid potentiating an existing crisis.

Mitigation strategies to the import of the brachytherapy source amidst the suspension of commercial airline services would be flight chartering but bureaucracy has been preventing implementation of this solution. Some of the reasons cited are associated with source transportation licensing and in country budget constraints.

There are lessons to be learnt though, including the exposure of the fragile systems we stand on to provide lifesaving cancer care to women in Zambia. We are positive some other nations, particularly lower and middle income countries (LMICs), may be at the brink of experiencing a similar dilemma because of the inability to import brachytherapy radioactive isotope sources and they too must act pre-emptively to avoid this senseless loss of life from a highly preventable disease.

## Conflicts of interest

The authors have no conflicts of interest to declare.

## Funding

This article did not receive any funding
